# An integrated physiology model to study regional lung damage effects and the physiologic response

**DOI:** 10.1186/1742-4682-11-32

**Published:** 2014-07-21

**Authors:** David A Shelley, Bryant L Sih, Laurel J Ng

**Affiliations:** 1 L-3 Applied Technologies, Inc., 10770 Wateridge Circle, Suite 200, San Diego, CA 92121, USA

**Keywords:** Mathematical modeling, Respiratory gas exchange, Physiology, Physical performance, Fatigue, Lung damage

## Abstract

**Background:**

This work expands upon a previously developed exercise dynamic physiology model (DPM) with the addition of an anatomic pulmonary system in order to quantify the impact of lung damage on oxygen transport and physical performance decrement.

**Methods:**

A pulmonary model is derived with an anatomic structure based on morphometric measurements, accounting for heterogeneous ventilation and perfusion observed experimentally. The model is incorporated into an existing exercise physiology model; the combined system is validated using human exercise data. Pulmonary damage from blast, blunt trauma, and chemical injury is quantified in the model based on lung fluid infiltration (edema) which reduces oxygen delivery to the blood. The pulmonary damage component is derived and calibrated based on published animal experiments; scaling laws are used to predict the human response to lung injury in terms of physical performance decrement.

**Results:**

The augmented dynamic physiology model (DPM) accurately predicted the human response to hypoxia, altitude, and exercise observed experimentally. The pulmonary damage parameters (shunt and diffusing capacity reduction) were fit to experimental animal data obtained in blast, blunt trauma, and chemical damage studies which link lung damage to lung weight change; the model is able to predict the reduced oxygen delivery in damage conditions. The model accurately estimates physical performance reduction with pulmonary damage.

**Conclusions:**

We have developed a physiologically-based mathematical model to predict performance decrement endpoints in the presence of thoracic damage; simulations can be extended to estimate human performance and escape in extreme situations.

## Background

Mathematical modeling in the area of human respiration is well established for healthy cases, often utilizing homogeneous lungs and uniform gas exchange. Some groups have developed models which account for lung heterogeneity; these models are typically either general and contain little geographical information (lobe/segment detail) [1-4] or are subject-specific and require a CT scan for full characterization [5-7]. Some groups have worked within a middle ground [8-10], however none of these modeling endeavors have incorporated ventilatory control, physical performance, and the impact of regional lung damage. Currently there is a need for a model which incorporates heterogeneous lungs with ventilatory control mechanisms, allowing for the prediction of how regional lung damage can impact gas exchange, respiratory control, and physical performance capabilities.

Pulmonary damage can be induced by external factors such as blast, blunt trauma and chemical injury [11-17]. Pulmonary damage has been quantified by measuring lung weight change or fluid infiltration (edema), which has been shown to impact gas exchange via shunting and altered diffusing capacity [18]. These processes reduce oxygen delivery to the blood, which can potentially reduce physical performance (time to fatigue) [19].

Our group has previously developed a dynamic physiology model (DPM) which predicts the ventilatory responses to oxygen-limiting environments, toxic gas inhalation and exercise [20,21]. The DPM ties together several independent modules into a single compartmentalized model with the ability to predict the physiological impact (e.g. ventilatory response, blood gas levels, gas exchange kinetics, and metabolism) from external stimuli (e.g. toxic gas exposure, exercise). The airway region is a steady-state two compartment approximation of the lungs which exchanges gas with the pulmonary capillaries. The DPM has been validated and shown to accurately account for the physiological response to several effectors. While the model accurately describes experimental data in healthy cases, the simplified airway compartment may not be adequate when accounting for heterogeneous lung behavior and damage.

The model developed here is an extension of the DPM which replaces the original steady-state airway compartment with a dynamic anatomic pulmonary system. The anatomic pulmonary model incorporates an asymmetric branching airway tree leading to independent lung segments with heterogeneous ventilation and perfusion, allowing for the simulation of regional and total lung damage. Lung injury via blunt trauma or blast damage leads to blood infiltration of the airways, an injury process known as edema. Edema can also be induced chemically with compounds that increase membrane permeability such as oleic acid. Edema from injury is modeled here as an additional barrier to gas transport, reducing the diffusing capacity across the alveolar membrane. Fluid infiltration also blocks oxygen delivery to damaged alveoli, resulting in a fraction of blood bypassing the site of oxygenation (shunt). For the purpose of gas exchange efficiency, the magnitude of lung injury is quantified in terms of fluid infiltration, which can be measured directly with water density changes on CT scans, or can be approximated post-mortem by physical measurements of lung weight and lung fluid levels. The lung injury model presented in this paper is calibrated with published data from animal experiments; injury mechanisms include blast, blunt trauma and oleic acid treatment. Adding this new level of detail into the previous model will allow for the prediction of physical performance impairment in the presence of heterogeneous lung damage.

## Methods

### Model development

**Figure 1:**
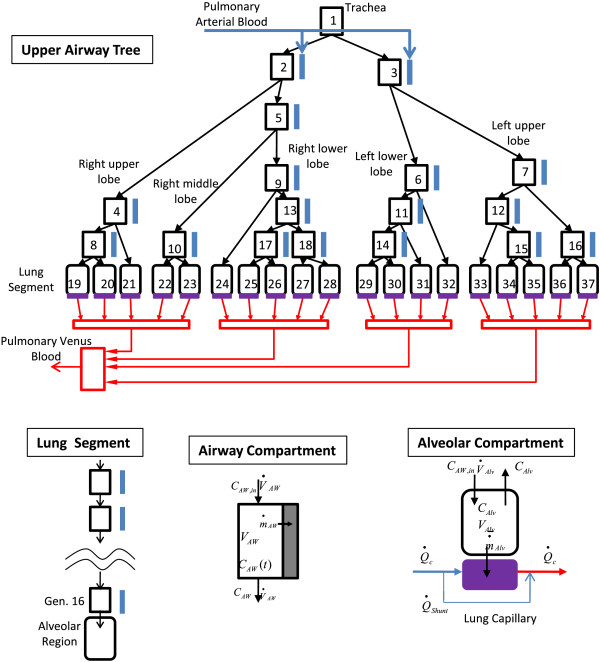
**Anatomic human pulmonary model.** Inspired gas enters the upper airway tree at the trachea and is delivered to the segmental compartments by a network of branching airway compartments. Each segmental compartment consists of a series of airway compartments leading to an alveolar compartment. The pulmonary circulation begins with the flow of deoxygenated blood from the heart; pulmonary arteries follow the path of the airway tree to the capillaries, where gas is exchanged with the alveolar regions. Oxygenated blood flows back to the heart via pulmonary veins.

The DPM contains two distinct pulmonary regions: the upper airway tree which is comprised of branching airway compartments, and the lung segments, which are comprised of a series of airway compartments and a terminal alveolar compartment (Figure 1). The model structure is designed to be able to account for damage at the segmental level that may result from any number of stressors including blast, blunt trauma, and chemical injury, and allows for predicting how regional or total lung damage can impact gas exchange. The new pulmonary model is designed to replace the steady-state two compartment airway region of the DPM, retaining all of the previously published features with the addition of a heterogeneous lung. Model equations derived below can be found in the Appendix.

### Upper airway tree

The upper airway tree spans from the trachea to the segmental bronchi (generations 0 to 3–6) and is composed of 18 compartments; compartmental organization and dimensions are based on Horsfield’s [22] asymmetric branching model #2. Each airway compartment is assumed to be well-mixed and can be modeled with an unsteady mass-balance which is applicable to any gas (Equation 1). V_AW,i_ is the volume of compartment *i* and is calculated using length (*l*_*i*_) and diameter (*d*_*i*_) measurements from the Horsfield model (Table 1). C_AW,i_ is the gas concentration in compartment *i* and $C_{\mathrm{AW},i}^{\mathrm{in}}$ is the inlet concentration. ${\overset{•}{V}}_{\mathrm{AW},i}$ is the volumetric flow through the compartment, while ${\overset{•}{m}}_{\mathrm{AW},i}$ is the gas uptake by diffusion through the airway wall. Because this study is mainly focused on the respiratory gases which do not exchange in the upper airways, this uptake term will be treated as negligible. Equation 1 can be used to describe both inhalation and exhalation by setting the source of $C_{\mathrm{AW},i}^{\mathrm{in}}$ to be the parent or daughter airway compartments, respectively.

**Table 1:** Human airway geometry

**Compartment**	**L (cm)**	**D (cm)**
1	100	16
2	22	11.1
3	50	12
4	15.6	7.3
5	26	8.9
6	11	8
7	16	7.5
8	6.4	8.5
9	8	6.4
10	21	5.2
11	18	6.5
12	14	7.3
13	8.4	6
14	4.5	7
15	13.5	5.3
16	11	5.5
17	6.2	3.2
18	14.8	6.2

### Lung segments

Each lung segment is separated into 2 regions; a conducting region which spans from the segmental bronchi (generation ~5) to the terminal bronchioles (generation 16) and an alveolar region which spans from generation 17 to 23. The conducting region is treated as a series of airway compartments representing the sum of the symmetric bifurcations at each generation. Each compartment can be characterized in the same way as the upper airway tree branches (Equation 1). The final airway compartment in each segment (generation 16) leads into the alveolar region for that segment. The alveolar regions are modeled using an unsteady mass-balance on a variable-volume chamber with gas being transported to or from the peripheral airways, and diffusion occurring across the capillary bed (Equation 2). V_Alv,i_ is the volume of alveolar compartment *i*, C_Alv,i_ is the gas concentration in alveolar compartment *i*, ${\dot{V}}_{\mathrm{Alv},i}$ is the volumetric flow into or out of the alveolar compartment and ${\dot{m}}_{\mathrm{Alv},i}$ is the mass transfer across the capillary membrane. Diffusion towards the capillaries is described by Fick’s first law (Equation 3), where D_L_ is the diffusing capacity for the entire membrane [23]. This can be scaled to describe transport in alveolar compartment *i* by the fractional membrane surface area in compartment *i*, f_i_ (Equation 4). Converting alveolar partial pressure (P_Alv,i_) to concentration (C_Alv,i_) and combining equations 2 and 4 gives the governing equation for alveolar concentration during inhalation and exhalation (Equation 5). For the case of inhalation, $\frac{dV_{\mathrm{Alv},i}}{\mathrm{dt}}={\dot{V}}_{\mathrm{Alv},i}$ and *C*_(*Alv*,*i*/*AW*,*i*)_ = *C*_*AW*,*i*_, reducing equation 2 to a time-dependent first order ordinary differential equation (ODE) (Equation 6). During exhalation, $\frac{dV_{\mathrm{Alv},i}}{\mathrm{dt}}={\dot{V}}_{\mathrm{Alv},i}$ and *C*_(*Alv*,*i*/*AW*,*i*)_ = *C*_*Alv*,*i*_, reducing equation 2 to another first order ODE (Equation 7).

### Lung capillaries

Each alveolar compartment exchanges gas through the alveolar membrane with an accompanying capillary region. For simplicity, the capillary network for a single segment is represented as one tube, where the exchange of gas along the length can be described by Fick’s first law (Equation 4), scaled by the fraction of surface area for diffusion in segment *k*, f_k_. Because capillary concentration is variable with respect to time and position, each tube was discretized into a series of compartments to yield a set of linear ODEs which could describe the time- and position-dependent capillary concentration profile (Equation 8). *P*_*c*,*i*_ and *C*_*c*,*i*_ are the gas partial pressure and concentration in capillary compartment *i*, respectively. *Q*_*c*,*i*_ is the capillary blood flow, *V*_*c*,*i*_ is the capillary volume, *n* is the number of serial divisions within the capillary tube, $C_{c,i}^{\mathrm{in}}$ is the inlet capillary concentration and *P*_*Alv*,*i*_ is the corresponding alveolar partial pressure. Capillary blood flow can be rewritten in terms of total blood flow, Q_Tot_, and segmental perfusion fraction, f_Q,k_ (Equation 9). The capillary volume in each serial compartment can be rewritten in terms of total capillary volume, V_c,tot_, segmental volume fraction, f_k_, and the number of serial capillary divisions, *n* (Equation 10). Equations 8 through 10 can be combined to yield an expression for the time-dependent concentration changes within any serial capillary compartment in any lung segment (Equation 11).

### Pulmonary veins and arteries

Morphological studies of the pulmonary circulation closely link each airway segment with an accompanying artery, while the venous network loosely follows the airways back to the heart [24-27]. To model these features, each airway compartment is matched with a parallel bronchial artery beginning at the left and right primary bronchus. The pulmonary arterial network follows the airway segments down to the capillary level, where gas exchange occurs (described above). The return of oxygenated blood to the heart is simplified in the model to 2 basic steps: capillary blood flow combines at the lobar level, then the lobar compartments combine as a feed to the systemic arteries [7,28].

The mechanism of a pulmonary shunt is incorporated into the model as a flow of blood at the capillary level that bypasses the site of gas exchange. Physically, this represents perfused lung tissue that is not taking part in gas exchange. In healthy subjects this flow is typically less than 5% of total cardiac output, however in disease states and following lung injury this can be increased [1,3,29,30].

### Sheep and goat models

**Figure 2:**
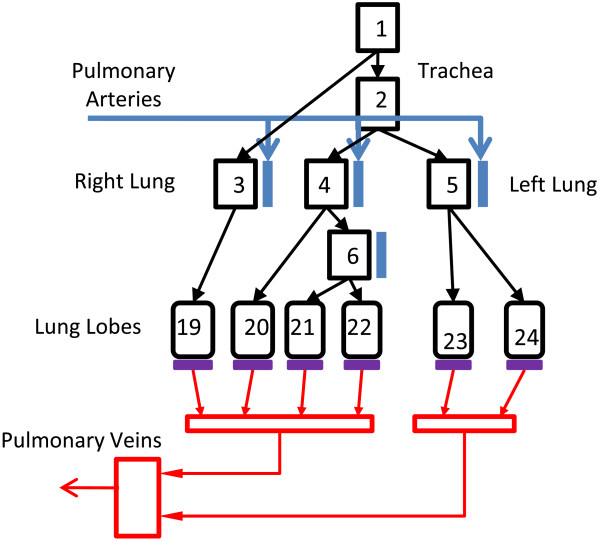
**Sheep lung model.** The sheep lung structure is modeled as an asymmetric bifurcating tree based on morphological measurements. The right lung contains 4 lobes, while the left lung contains 2 lobes. The pulmonary arteries track the airways to the capillary bed, where the blood is oxygenated and returns to the systemic circulation via pulmonary veins.

Due to the lack of human lung injury data, the DPM was expanded to be applicable to several animal species. A majority of lung injury experiments in the literature utilize sheep and goats, both of which have similar lung structures and will be the focus of the animal model injury calibration process. The sheep lung is composed of 6 lobes, the left apical and diaphragmatic lobes, and the right apical, diaphragmatic, accessory and middle lobes [31,32]. The model structure of the sheep airway tree was designed using the same method previously described for humans. The asymmetric branching pattern for sheep is based on morphometric measurements reported in the literature [31,33,34]. The sheep model includes asymmetric branching from the trachea to the lobes; with a single distal airway compartment emptying into a single alveolar compartment for each lobe (Figure 2). Compartmental dimensions are presented in Table 2. This lobar structure provides less regional detail than the human segmental model, but is necessary due to fewer available morphometric sheep studies. Non-pulmonary compartments within the DPM (systemic circulation, tissue, brain) have been previously validated for animals and remain unchanged.

**Table 2:** Sheep model dimensions

**Compartment**	**Length [mm]**	**Volume [ml or% Alveolar]**
1	91.1	8.6
2	40	3.8
3	8.2	0.05
4	15	0.29
5	16	0.36
6	8.2	0.05
19	-	12.1%
20	-	31.1%
21	-	4.9%
22	-	9.4%
23	-	13.5%
24	-	29.1%

### Ventilation and perfusion

Inhalation and exhalation flows can be determined based on respiratory frequency (Freq) and tidal volume (VT) with the assumption that on average 35% of the breathing cycle occurs during inspiration and 65% during exhalation [27]. The fractional alveolar flows in each of the lung segments can be approximated using the segment height from the base of the lung. West [35] identified a linear relationship between height within the lung and ventilation rate, where the basal regions had proportionally higher ventilation rates. This relationship is described by Equation 12, where *h* is lung height (% from base) and $\dot{V}$ is ventilation rate normalized by percent lung volume. Using average segment height and volume from CT scans (Table 3), fractional alveolar ventilation flows can be approximated. Capillary perfusion can also be described as a function of height within the lung by Equation 13 [35]. The fraction of total cardiac output entering the capillaries in a single alveolar region can be calculated based on mean segment height and volume (Table 3).

**Table 3:** Human lung segment dimensions

**Lung segment**	**Volume (% of total)**	**Height (% from base)**
19	6.4	76.1
20	5.5	69
21	6.7	89
22	3.4	39
23	4	22.3
24	5.8	35.9
25	4.3	0
26	5.1	2.3
27	5.3	9.8
28	4.8	16.1
29	7.3	14
30	6.2	6.4
31	8.4	6.2
32	7.4	41.3
33	4.2	78.2
34	4	1
35	4	94
36	4.1	51.9
37	3.1	40.6

### Pulmonary damage

Pulmonary damage is modeled as fluid/blood infiltration in the airways. Fluid infiltration reduces the diffusing capacity for oxygen and is accounted for in the model as a percent reduction in D_L_. A pulmonary shunt is accounted for in the model as a percent of blood flow that bypasses the oxygenation process. The shunted blood recombines with the oxygenated blood before entering the systemic circulation. A mass balance of the lung capillaries (Equation 8) highlights the impact of both the D_L_ reduction and the Q_c,i_ bypass, both of which impair oxygenation. Damage level due to fluid infiltration is considered to be steady-state for model simulations. Physiological predictions are generated for a fixed damage level with the assumption that injury has reached steady state and simulation time is less than recovery time.

### Model solution

Governing equations were solved using Matlab/Simulink. An implicit solution algorithm was utilized for the airway and capillary compartments to achieve numerical stability [36]. Alveolar equations were left in differential form and solved using the built-in integration tool in Simulink.

### Parameter estimation and scaling

As described above, airway dimensions were incorporated from the Horsfield Model [22], while ventilation and perfusion of each segment were estimated based on data from West [35]. The alveolar diffusing capacities for oxygen (40 ml/mmHg-min) and carbon dioxide (800 ml/mmHg-min) were based on the data of Hill [37], The alveolar oxygen diffusing capacity was assumed to be a function of oxygen partial pressure and workload as described by Graeser [38] (Equation 15), where $P_{\mathrm{Alv},O_{2}}$ is alveolar oxygen (mmHg) and Work is the exercise load in Watts. All DPM parameters outside of the pulmonary system are modeled as previously described [20,21]. The airway volumes measured by Horsfield represent an average human. As reported by Lindstedt [39], volumes can be scaled linearly with body mass. To maintain similar structure, surface areas were scaled by body mass to the 2/3 power. Scaling in the non-airway compartments of the DPM has been previously described [20,21].

## Results

### Ventilatory response to incremental exercise

**Figure 3:**
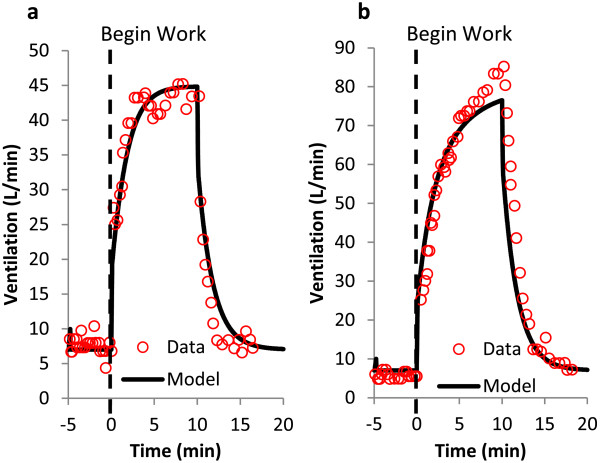
**Ventilatory response to incremental exercise.** Ventilatory response to a step increase in workload from rest on a treadmill. **(a)** Step increase to workload corresponding to 50% of VO2,max. **(b)** Step increase to workload corresponding to 80% of VO2,max. Data from Mateika and Duffin [40]. Experimental data presented as circles, model prediction is a solid line.

Mateika and Duffin [40] studied the effects of abrupt step changes in exercise workload on a treadmill in normoxic conditions. Subjects ran at intensities that produced 50% and 80% maximal oxygen consumption (V_O2,max_) (Figure 3). Prior to the start of exercise, subjects stood on the treadmill for 3 to 5 minutes; at the end of exercise the treadmill was stopped abruptly. Ventilation was recorded throughout the experiment. The DPM is able to simulate the rapid increase in ventilation to a new steady state value, as well as the exponential decrease upon completion of exercise.

### Physiological response to hypoxia exposure at rest

**Figure 4:**
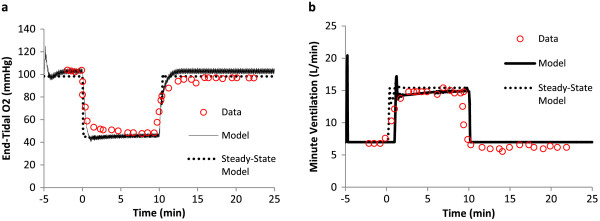
**Physioligal response to hypoxia exposure at rest.** Ventilatory **(a)** and Alveolar PO2 **(b)** response to step change from room air to 9% O2 for 10 minutes, followed by a return to room air. Alveolar CO2 is held constant via external supply (isocapnic hypoxia). Data from Reynolds [41]; experimental data presented as circles, model prediction is a solid line. Experimental alveolar O2 is approximated to be equal to end-tidal O2.

Reynolds and Milhorn [41] studied the ventilation response due to hypoxia. Subjects were exposed to 10 minutes of acute isocapnic hypoxia. O_2_ was held at 9% over the course of exposure, while a steady alveolar CO_2_ concentration was maintained. After 10 minutes, oxygen levels were returned to normal. At the start of hypoxic exposure, end-tidal oxygen levels rapidly decreased, reaching a plateau around 5 minutes (Figure 4a). O_2_ levels returned to normal following the conclusion of hypoxic exposure; steady state was achieved within a few minutes of normoxia. The rapid reduction in oxygen saturation during hypoxia leads to an increase in ventilation which overshoots the final steady state plateau. This overshoot is a result of the ventilation control equations, which are driven by changes in O_2_ saturation.. An exponential decay in ventilation is seen from the initial overshoot to the final plateau within minutes of exposure (Figure 4b). Upon returning to normoxia, the ventilation dropped to baseline levels. This augmented DPM is able to reproduce these trends in alveolar oxygen concentration and ventilation (solid black line). Results from the previous version of the DPM with steady-state airways are also included (dashed black line) to highlight model differences. The steady-state lung response to hypoxia is faster than the new dynamic model, which lags due to an alveolar wash-out period. This lag also leads to a lag in ventilation increase with hypoxia, which is a function of O_2_ saturation.

### Physiological response to hypoxia exposure during exercise

**Figure 5:**
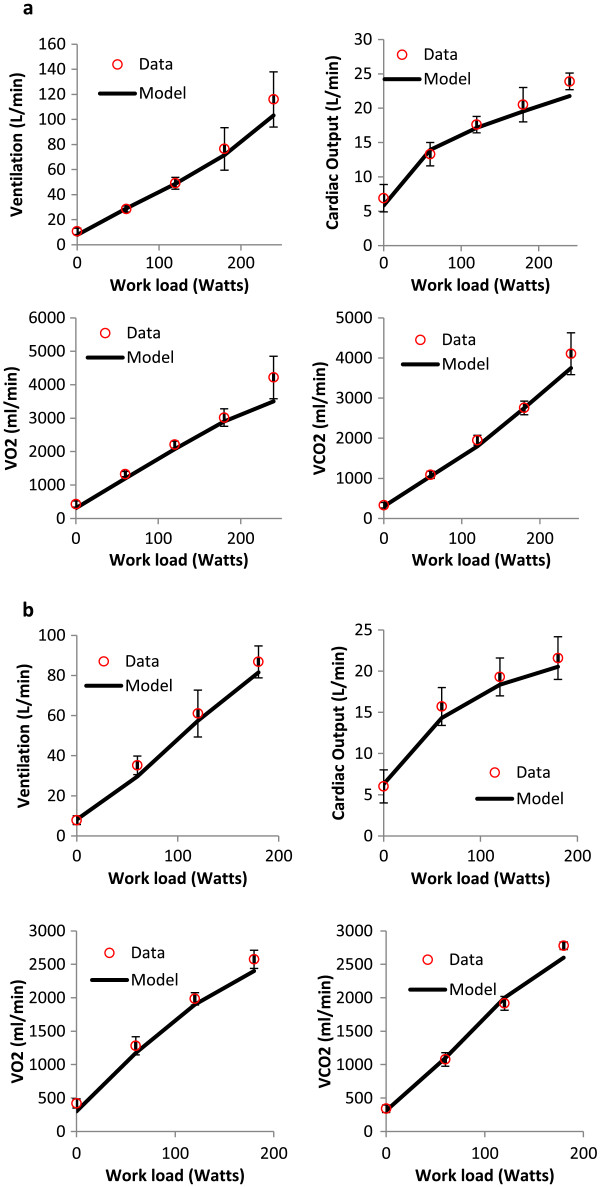
**Physiological response to hypoxia exposure during exercise.** Model predictions (line) and experimental data (circles) for physiological variables in response to various workloads at sea level (normoxia) and a simulated altitude of 429 mmHg, corresponding to 11.85% Oxygen (hypoxia). Workloads ranged from 0 to 240 W; physiological measurements were made at steady-state. Data from Wagner [42].

Wagner [42] studied the steady-state physiological response to exercise in normoxia and hypoxia. Each subject cycled at a constant workload for 5 to 10 minutes to allow the body to reach steady state. Workloads spanned from 0 to 240 Watts. At steady state, the researchers measured several physiological variables including ventilation, blood flow, VO_2_, and VCO_2_. The experiment was performed at normoxia and simulated altitude (hypoxia, 11.85% O_2_). Experimental results and model predictions are plotted as a function of exercise load for normoxia and hypoxia (Figure 5). The model predictions fall within experimental ventilation and blood flow for both normoxic and hypoxic conditions. VO_2_ and VCO_2_ match the data with a slight under prediction at the highest work due to a model ventilation that was just below the average value at this activity level. This under-prediction is due to the ventilation prediction being slightly lower than average for extreme workloads.

### Oleic acid damage in goats with exercise

**Figure 6:**
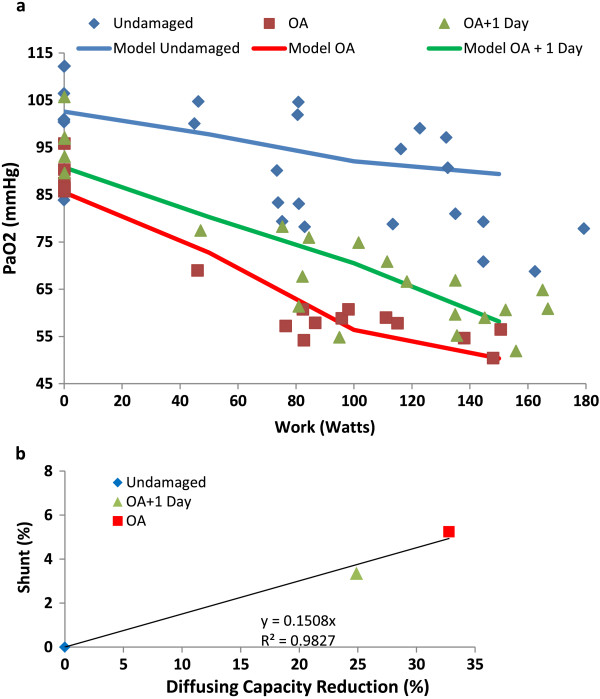
**Oleic acid damage in goats with exercise. (a)** Steady-state arterial O2 measured in exercising goats [43]. Conditions include healthy (diamonds), oleic acid treated (squares) and oleic acid with 1 day recovery (triangles). Solid lines are model fit. **(b)** Model shunt and diffusing capacity damage required to reproduce exercise data. Diamond is undamaged, triangle is OA + 1 day recovery and square is OA. Solid line represents best-fit of data.

Goats were treated with oleic acid (OA) and run on a treadmill in Crocker et al. [43]. Runs were done at 3 speeds for each goat under healthy conditions, OA treatment and following 1 day of recovery from OA. All measurements were done at steady-state, and included physiologic values such as arterial and venous oxygen saturation and CO2 levels, respiratory rate, temperature, and heart rate. OA treatment is assumed to induce uniform lung damage via permeability edema. Plotting arterial O_2_ versus workload (Figure 6a) yields 3 curves; undamaged, OA treated and one day recovery from OA. D_L_ reduction and shunt were simultaneously fit in the DPM to the 3 experimental curves to develop a relationship between the response of D_L_ and shunt to different degrees of lung damage. The model was fit to experimental data by varying shunt and DL to minimize the least squares error with the data. Plotting the required shunt against D_L_ yields a linear relationship (Figure 6b). The predicted behavior of percent shunt and D_L_ reduction with increasing damage is assumed to be uniform across the whole lung for oleic acid induced permeability edema, and roughly follows equation 16.

### Pulmonary damage dose response

**Figure 7:**
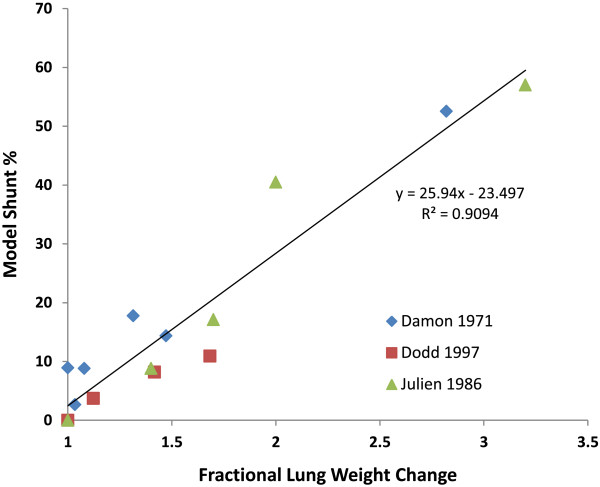
**Pulmonary damage dose response.** The required model shunt to account for steady-state arterial oxygen concentration changes in experimental damage plotted as a function of fractional lung weight change. Damon et al. (diamonds) reports values for individual sheep placed sheep in shock tubes. Dodd et al. (squares) reports mean values for groups of sheep in response to 3 shock tube damage levels. Julien et al. (triangles) measured the dose response of oleic acid treatment.

Julien et al. [13] utilized sheep to develop a dose response curve of arterial O_2_ to a range of OA treatment concentrations. For each dose of OA, steady-state values of arterial oxygen were recorded. Following measurement, the animals were sacrificed to determine lung water content. Changes in lung water were converted to estimated lung weight increases using relationships previously published by the same group [44,45]. Maintaining the relationship between shunt and D_L_ derived from the OA exercise data (Equation 16), the required model shunt was estimated to match observed drops in arterial O_2_ at each OA dose. The model shunt is plotted against experimental lung weight change in Figure 7 (triangles). As experimental lung weight increases with blood infiltration, the model damage parameters also increase to account for the observed reduction in arterial oxygen.

### Pulmonary damage in sheep from shock tube

Damon [14] measured the effects of shock tube injury on resting sheep. The animals were placed inside a large shock tube with left side pressed against the end plate. Pulmonary function testing was performed before and after injury; measurements included arterial gas concentrations and ventilatory parameters. Post-mortem determination of lung weight change with injury was determined following the final pulmonary test. For each set of pre- and post-shock data, the DPM was used to determine the required model shunt and D_L_ reduction to account for the reduction in arterial O_2_. Model shunt is plotted against experimental lung weight increase (Figure 7, diamonds) for each animal. As with the OA studies, increasing damage (fluid infiltration) requires increasing pulmonary shunt within the DPM. Dodd [11] performed similar studies on sheep with a small shock tube placed against the left side of the animal. Measurements were made of arterial O_2_ before and after blast, as well as post-mortem lung weights. Data is presented as the mean values for a group of sheep at each damage level (0, 1, 2 and 3). Utilizing the relationship between shunt and D_L_ previously established, the required shunt for each case was plotted versus lung weight change (Figure 7, squares). The data follow a similar trend to the previous studies with OA and shock injury in sheep.

### Fatigue runs with pulmonary damage in sheep

**Figure 8:**
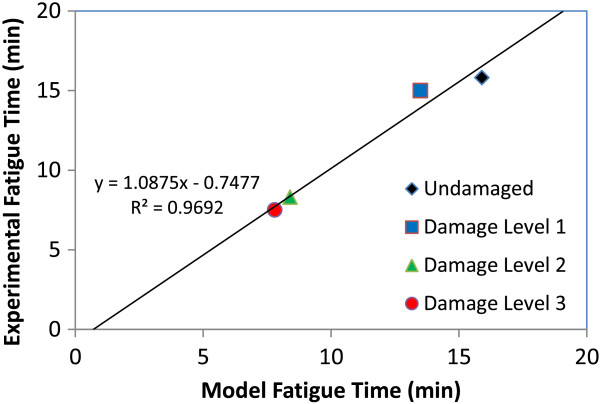
**Fatigue time in sheep with lung injury.** Experimental vs model predicted time to fatigue for ramping exercise and increasing damage levels. Control sheep and shock tube damaged sheep were run to fatigue on a treadmill. Runs began at 0.67 m/s and increased by 0.22 m/s every 90 seconds until the sheep fatigued. Run speed was held constant once reaching 2.22 m/s. The model predictions are based on the muscle fatigue module in the DPM, as described previously [20].

Januszkiewicz [19] and Mundie [46] followed the same shock tube protocol described above, while extending the study to measure changes in time to fatigue in sheep as a function of damage level. Sheep were exposed to 3 levels of damage and subsequently run on a treadmill at 9% grade. Runs began at 0.67 m/s and increased by 0.22 m/s every 90 seconds until the sheep fatigued. Run speed was held constant once reaching 2.22 m/s. Using the work profile as the DPM input, and maintaining the relationship between shunt and D_L_ previously established, simulated fatigue runs were generated for each damage condition. The model damage parameters (shunt and D_L_) were set using the fit of Dodd’s shock tube data above. Muscle fatigue parameters were fit to the data, optimizing the 4 different fatigue times simultaneously. Experimental fatigue time and model predicted fatigue time are plotted (Figure 8). The DPM is able to produce the trends seen experimentally; time to fatigue decreases with increasing pulmonary damage.

## Discussion

The development and integration of an anatomic pulmonary model into the previously described DPM allows for the possibility of simulating partial or total lung damage and relating that damage to gas exchange and subsequent oxygen delivery to the body. Thus, the DPM fills a gap in current modeling capabilities, allowing for the estimation of the impact of regional lung damage on oxygen and carbon dioxide exchange. The model can be used to understand the impact of pulmonary damage on physical performance.

With the introduction of the new pulmonary module, the first task is to confirm the model maintains the ability to simulate the physiologic responses described in previous reports. The exercise response calculations utilized in the previous model are unchanged in the current version; workload and muscle movement directly impact tidal volume and frequency as described previously [20]. Consequently, the ventilatory response to exercise agrees well with the literature data which the original exercise model was based upon (Figures 3 and 4). The physiological response to exercise and hypoxia also remains accurate (Figure 5). Thus, the introduction of the anatomic pulmonary module does not alter the previously validated response; the same amount of oxygen reaches the alveolar and capillaries in the same time-frame as the data suggests.

While the model in this paper is the first model to couple heterogeneous geographical information (segments and lobes) with ventilation control models, previous groups have developed independent lung models to examine the effects of airway heterogeneity. Tang et al. [3] utilized a 15-compartment model of the airways with symmetric branching based on the Weibel data. This model is capable of simulating pulmonary shunts, as well as ventilation/perfusion mismatches by modeling the alveolar region as 4 compartments with unique ventilation/perfusion ratios. While this model allows for unsteady breath-to-breath simulations of gas exchange, as well as simulated dysfunction via the shunt and V/Q distributions, there is no specific regional information included. This could lead to difficulties in expanding the model to blunt trauma and blast damage, where the geographical location and relative magnitude of damage can be predicted by finite element models. Additional models have been developed by Yem [1,2] and Whiteley [4] with similar features and drawbacks. Gisolf [47] developed a model which introduced some level of geographical information by assigning nine ventilation/perfusion compartments as parallel vertical slices of the human lung. This model structure allows for more relevant geographical studies in damage and gas exchange. Regardless, no previous model couples the heterogeneous lung with an ability to predict physiologic response to regional damage, as well as ventilation control under exercise, toxic gas exposure and reduced oxygen supply. The integration of pulmonary damage into the DPM allows for the ability to predict the effects of regional lung damage on physical performance endpoints. Lung damage from blunt trauma, blast or chemical injury is modeled as fluid infiltration of the airways; increased fluid levels decreases the diffusing capacity for oxygen transport and can result in blood bypassing the active site of oxygenation. The introduction of regional pulmonary damage allows for the model to be used to simulate human performance in response to stressors including exposure to low oxygen, toxic gas, exercise and heterogeneous thoracic trauma.

Estimating the impact of fluid infiltration on shunt and diffusing capacity is accomplished by examining pulmonary function data from animals treated with oleic acid. OA causes permeability edema and is assumed to act uniformly on the lungs. The model was used to predict the response to damage in OA treated exercising goats. Measurements were taken at baseline, following OA treatment and after 1 day of recovery, giving 3 separate damage conditions. For each case, the model best-fit curve was generated by treating shunt and diffusing capacity as variables. Assuming the lung damage was uniform and steady-state at the time of measurement, a curve was established relating D_L_ and shunt changes to changes in damage level. With limited exercise data for only 3 injury conditions, the estimated relationship between experimental damage level and model damage parameters would benefit from additional data. The linear trend linking shunt and D_L_ should be further explored when more data becomes available; further experiments are also required to understand how the relationship behaves at more extreme damage levels. For the current study, the developed relationship between D_L_ and shunt changes to damage level is assumed to be true in applying damage to the whole lung, or to regional damage such as blast or blunt trauma.

Utilizing this relationship between experimental damage and model damage parameters, shunt and D_L_ changes were then linked with experimental lung weight increases from fluid infiltration in sheep following either OA treatment or blast damage. This link allows for quantitative prediction of the effects of regional damage if regional lung weight change is known. The required model shunt was plotted against lung weight increase (Figure 7) for three separate experiments. The combined model predictions for each experiment yields a linear trend between lung weight increase and the required damage values. This correlation is important, as it allows for linking physical performance in the DPM to blast and blunt trauma damage-prediction algorithms that output lung weight increase estimates such as those developed by MacFadden et al. [48]. This allows for simulation of performance decrement in the presence of complex blast patterns or blunt trauma behind armor. The model has been utilized to predict performance decrement in sheep blasted with shock tubes (Figure 8). The experimental fatigue time aligns with the model predicted fatigue times for all damage levels, and produces a line close to identity.

**Figure 9:**
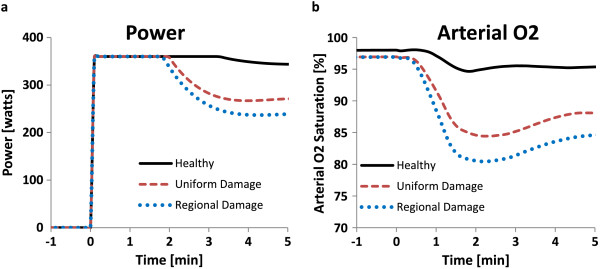
**Predicted exercise response to lung damage patterns.** Theoretical human response to step-increase in workload (360 Watts at time = 0) with uniform or regional lung damage. Simulations were run for 3 conditions: healthy, uniform damage, and regional damage. Uniform damage was simulated as 20% lung weight gain across all segments. Regional damage represents the same total lung weight gain as uniform damage, confined to the right lung segments only (38.1% weight gain in right lung, calculated using volume fraction of right lung to total lung volume). **(a)** Workload output under each condition; deviation from the desired workload (360 watts) represents fatigue time. **(b)** Arterial O_2_ saturation profile for each condition.

The model can be used to predict the impact of regional vs. uniform lung damage on human exercise capabilities (Figure 9). Simulations were run to compare the difference in oxygenation and time-to-fatigue following a step increase in workload (360 watts) for the case of uniform lung damage, and an equivilant amount of regional lung damage. Uniform damage was simulated as 20% lung weight gain across all segments. Regional damage was simulated by lumping all of the damage from the uniform case into the right lung only, which was calculated by scaling the total weight gain percent (20%) by the fraction of right lung volume to total lung volume (0.52), resulting in a weight gain of 38.1% in each of the right lung segments. Simulated power output (Figure 9a) demonstrates that the damage leads to earlier fatigue than a healthy case (deviation from desired workload of 360 Watts). Regional damage conditions fatigue slightly faster than uniform damage due to the reduced oxygenation (Figure 9b). This reduced oxygenation in moderate regional damage vs. mild uniform damage is likely due to the alveolar-capillary diffusion limitations in exercise with high blood flow and increased O_2_ consumption.

There are limitations to the predictive power of the DPM in terms of response to extreme conditions. The underlying models were derived and validated for several environmental conditions and exercise levels. However, the human response at extremes (e.g. maximum sprinting speed) can be highly variable between subjects. Additionally, the DPM is designed to simulate the average human response under healthy conditions. This paper introduced the ability to describe pulmonary damage, however the DPM still represents the response of a healhy human (e.g. not accurate for disease states).

## Conclusion

With the development of the DPM, we have built a unique tool which utilizes a heterogeneous pulmonary model and predicts physical performance decrement in response to stimuli such as exercise, toxic gas exposure, hypoxia, blunt trauma, blast damage and chemical lung injury. The DPM is validated against experimental data, predicting physiologic response to exercise and hypoxia in humans. The damage parameters were calibrated using available animal lung damage experiments; shunt and diffusing capacity reduction caused by fluid infiltration is correlated to experimental lung weight change. The resulting model is capable of predicting physical performance decrement in response to a range of stressors.

## Appendix

(1) $$V_{\mathrm{AW},i}\frac{dC_{\mathrm{AW},i}}{\mathrm{dt}}={\dot{V}}_{\mathrm{AW},i}\left(C_{\mathrm{AW},i}^{\mathrm{in}}-C_{\mathrm{AW},i}\right)-{\dot{m}}_{\mathrm{AW},i}$$

(2) $$V_{\mathrm{Alv},i}\frac{dC_{\mathrm{Alv},i}}{\mathrm{dt}}+C_{\mathrm{Alv},i}\frac{dV_{\mathrm{Alv},i}}{\mathrm{dt}}={\dot{V}}_{\mathrm{Alv},i}C_{\left(\mathrm{Alv},i/\mathrm{AW},i\right)}-{\dot{m}}_{\mathrm{Alv},i}$$

(3) $${\dot{m}}_{\mathrm{Alv}}=D_{L}\left(P_{\mathrm{Alv}}-P_{c}\right)$$

(4) $${\dot{m}}_{\mathrm{Alv},i}=f_{i}D_{L}\left(P_{\mathrm{Alv},i}-P_{c,i}\right)$$

(5) $$V_{\mathrm{Alv},i}\frac{dC_{\mathrm{Alv},i}}{\mathrm{dt}}+C_{\mathrm{Alv},i}\frac{dV_{\mathrm{Alv},i}}{\mathrm{dt}}={\dot{V}}_{\mathrm{Alv},i}C_{\left(\mathrm{Alv},i/\mathrm{AW},i\right)}-f_{i}D_{L}\left(\mathrm{RT}C_{\mathrm{Alv},i}-P_{c,i}\right)$$

(6) $$V_{\mathrm{Alv},i}\frac{dC_{\mathrm{Alv},i}}{\mathrm{dt}}={\dot{V}}_{\mathrm{Alv},i}\left(C_{\mathrm{AW},i}-C_{\mathrm{Alv},i}\right)-f_{i}D_{L}\left(\mathrm{RT}C_{\mathrm{Alv},i}-P_{c,i}\right)$$

(7) $$V_{\mathrm{Alv},i}\frac{dC_{\mathrm{Alv},i}}{\mathrm{dt}}=-f_{i}D_{L}\left(\mathrm{RT}C_{\mathrm{Alv},i}-P_{c,i}\right)$$

(8) $$V_{c,i}\frac{dC_{c,i}}{\mathrm{dt}}=Q_{c,i}\left(C_{c,i}^{\mathrm{in}}-C_{c,i}\right)+\frac{f_{k}D_{L}}{n}\left(P_{\mathrm{Alv},i}-P_{c,i}\right)$$

(9) $$Q_{c,i}=Q_{\mathrm{Tot}}f_{Q,k}$$

(10) $$V_{c,i}=\frac{V_{\mathrm{capillary}}f_{k}}{n}$$

(11) $$\frac{dC_{c,i}}{\mathrm{dt}}=\frac{nQ_{\mathrm{Tot}}f_{Q,k}}{V_{\mathrm{capillary}}f_{k}}\left(C_{c,i}^{\mathrm{in}}-C_{c,i}\right)+\frac{D_{L}}{V_{\mathrm{capillary}}}\left(P_{\mathrm{Alv},i}-P_{c,i}\right)$$

(12) $$\dot{V}=-0.00031*h+0.064$$

(13) $$\overset{•}{Q}=-0.0009*h+0.1$$

(14) $$V_{c,i}\frac{dC_{c,i}}{\mathrm{dt}}=Q_{c,i}\left(C_{c,i}^{\mathrm{in}}-C_{c,i}\right)+\frac{f_{k}D_{L}}{n}\left(P_{\mathrm{Alv},i}-P_{c,i}\right)$$

(15) $$D_{L,O_{2}}=-0.0078P_{\mathrm{Alv},O_{2}}^{2}+\left(0.3+\frac{\mathrm{Work}}{833}\right)P_{\mathrm{Alv},O_{2}}+\left(76+\frac{\mathrm{Work}}{6}\right)$$

(16) $$\mathrm{Shunt}\approx 0.15*D_{L,\mathrm{reduction}}$$

## Abbreviations

β: Gas solubility; C: Gas concentration; D: Diffusion coefficient; D_AW,j_: Airway diffusing capacity; D_L_: Alveolar diffusing capacity; f: Fraction of total alveolar volume; f_Q_: Perfusion fraction; Freq: Respiratory rate; h: Lung segment height; l: Thickness of diffusion layer; $\dot{m}$: Mass uptake; n: Number of capillary divisions; p: Gas partial pressure; Q: Blood flow; Q_Tot_: Total cardiac output; R: Gas-constant; T: Temperature; V: Volume; $\dot{V}$: Flow rate; V_capillary_: Total lung capillary volume; V_T_: Tidal volume.; Alv: Alveolar region; AW: Airway region; b: Blood; c: Capillary region; E: Exhalation; in: Inlet value; I: Inhalation; i: Compartment i; j: Gas j; k: Segment k; m: Airway mucus layer; t: Airway tissue layer.

## Competing interests

The authors declare that they have no competing of interests.

## Authors’ contributions

DS developed the model and completed the manuscript. BS aided in physical performance calculations and provided technical expertise. LN provided technical expertise and aided in manuscript preparation. All authors read and approved the final manuscript.

## References

[B1] Yem JS Turner MJ Baker AB Young IH Crawford AB A tidally breathing model of ventilation, perfusion and volume in normal and diseased lungs Br J Anaesth 2006 97 5 718 731 10.1093/bja/ael216 16926169

[B2] Yem JS Tang Y Turner MJ Baker AB Sources of error in noninvasive pulmonary blood flow measurements by partial rebreathing: a computer model study Anesthesiology 2003 98 4 881 887 10.1097/00000542-200304000-00014 12657849

[B3] Tang Y Turner MJ Baker AB Effects of alveolar dead-space, shunt and V/Q distribution on respiratory dead-space measurements Br J Anaesth 2005 95 4 538 548 10.1093/bja/aei212 16126784

[B4] Whiteley JP Gavaghan DJ Hahn CE A tidal breathing model of the inert gas sinewave technique for inhomogeneous lungs Respir Physiol 2001 124 1 65 83 11084204 10.1016/s0034-5687(00)00185-7

[B5] Tawhai HM Pullan AJ Hunter PJ Generation of an anatomically based three-dimensional model of the conducting airways Ann Biomed Eng 2000 28 7 793 802 11016416 10.1114/1.1289457

[B6] Tawhai MH Hunter P Tschirren J Reinhardt J McLennan G Hoffman EA CT-based geometry analysis and finite element models of the human and ovine bronchial tree J Appl Physiol 2004 97 6 2310 2321 10.1152/japplphysiol.00520.2004 15322064

[B7] Burrowes KS Hunter PJ Tawhai MH Anatomically based finite element models of the human pulmonary arterial and venous trees including supernumerary vessels J Appl Physiol 2005 99 2 731 738 10.1152/japplphysiol.01033.2004 15802366

[B8] Arieli R Farhi LE Gas exchange in tidally ventilated and non-steadily perfused lung model Respir Physiol 1985 60 3 295 309 10.1016/0034-5687(85)90059-3 4035107

[B9] Busso T Robbins PA Evaluation of estimates of alveolar gas exchange by using a tidally ventilated nonhomogenous lung model J Appl Physiol 1997 82 6 1963 1971 9173965 10.1152/jappl.1997.82.6.1963

[B10] Van EC Hirsch C Paiva M Anatomically based three-dimensional model of airways to simulate flow and particle transport using computational fluid dynamics J Appl Physiol 2005 98 3 970 980 15501925 10.1152/japplphysiol.00795.2004

[B11] Dodd KT Mundie TG Lagutchik MS Morris JR Cardiopulmonary effects of high-impulse noise exposure J Trauma 1997 43 4 656 666 10.1097/00005373-199710000-00016 9356064

[B12] Liu B Wang Z Leng H Yang Z Li X Studies on the mechanisms of stress wave propagation in the chest subjected to impact and lung injuries J Trauma 1996 40 3 Suppl S53 S55 8606422 10.1097/00005373-199603001-00011

[B13] Julien M Hoeffel JM Flick MR Oleic acid lung injury in sheep J Appl Physiol 1986 60 2 433 440 3949648 10.1152/jappl.1986.60.2.433

[B14] Damon EG Yelverton JT Luft UC Mitchell K Jr Jones RK Acute effects of Air blast on pulmonary function in dogs and sheep Aerosp Med 1971 42 1 1 9 5541085

[B15] Argyros GJ Management of primary blast injury Toxicology 1997 121 1 105 115 10.1016/S0300-483X(97)03659-7 9217319

[B16] Phillips YY Primary blast injuries Ann Emerg Med 1986 15 12 1446 1450 10.1016/S0196-0644(86)80940-4 3535591

[B17] Hedlund LW Effmann EL Bates WM Beck JW Goulding PL Putman CE Pulmonary edema: a CT study of regional changes in lung density following oleic acid injury J Comput Assist Tomogr 1982 6 5 939 946 10.1097/00004728-198210000-00012 7142509

[B18] Chiang CH Shen CY Hsu K Correlation between cardiopulmonary changes and severity of acute lung injury in dogs Crit Care Med 1990 18 4 419 422 10.1097/00003246-199004000-00014 2108003

[B19] Januszkiewicz AJ Mundie TG Dodd KT Maximal exercise performance-impairing effects of simulated blast overpressure in sheep Toxicology 1997 121 1 51 63 10.1016/S0300-483X(97)03655-X 9217315

[B20] Ng LJ Sih BL Stuhmiller JH An integrated exercise response and muscle fatigue model for performance decrement estimates of workloads in oxygen-limiting environments Eur J Appl Physiol 2012 112 4 1229 1249 10.1007/s00421-011-2062-5 21769737

[B21] Stuhmiller JH Stuhmiller LM A mathematical model of ventilation response to inhaled carbon monoxide J Appl Physiol 2005 98 6 2033 2044 10.1152/japplphysiol.00034.2005 15691907

[B22] Horsfield K Dart G Olson DE Filley GF Cumming G Models of the human bronchial tree J Appl Physiol 1971 31 2 207 217 5558242 10.1152/jappl.1971.31.2.207

[B23] Bird RB Stewart WE Lightfoot EN Transport phenomena 2007 New York: J. Wiley

[B24] Burrowes KS Swan AJ Warren NJ Tawhai MH Towards a virtual lung: multi-scale, multi-physics modelling of the pulmonary system Philos Transact A Math Phys Eng Sci 2008 366 1879 3247 3263 10.1098/rsta.2008.0073 PMC3268218 18593661

[B25] Horsfield K Morphometry of the small pulmonary arteries in man Circ Res 1978 42 5 593 597 10.1161/01.RES.42.5.593 639181

[B26] Singhal S Henderson R Horsfield K Harding K Cumming G Morphometry of the human pulmonary arterial tree Circ Res 1973 33 2 190 197 10.1161/01.RES.33.2.190 4727370

[B27] Seeley RR Stephens TD Tate P Anatomy & Physiology 2003 Boston: Mass.: McGraw-Hill

[B28] Huang W Yen RT McLaurine M Bledsoe G Morphometry of the human pulmonary vasculature J Appl Physiol 1996 81 5 2123 2133 8941537 10.1152/jappl.1996.81.5.2123

[B29] Batchinsky AI Weiss WB Jordan BS Dick EJ Jr Cancelada DA Cancio LC Ventilation-perfusion relationships following experimental pulmonary contusion J Appl Physiol 2007 103 3 895 902 10.1152/japplphysiol.00563.2006 17569766

[B30] Wagner PD Laravuso RB Uhl RR West JB Continuous distributions of ventilation-perfusion ratios in normal subjects breathing air and 100 per cent O2 J Clin Invest 1974 54 1 54 68 10.1172/JCI107750 4601004 PMC301524

[B31] Hare WC The broncho-pulmonary segments in the sheep J Anat 1955 89 3 387 402 13251969 PMC1244767

[B32] Walther SM Domino KB Glenny RW Polissar NL Hlastala MP Pulmonary blood flow distribution has a hilar-to-peripheral gradient in awake, prone sheep J Appl Physiol 1997 82 2 678 685 9049752 10.1152/jappl.1997.82.2.678

[B33] Robinson SM Cadwallader JA Hill PM An animal model for the study of regional lung function J Appl Physiol 1978 45 2 320 324 681217 10.1152/jappl.1978.45.2.320

[B34] Lipsett J Analysis of the conducting airway system in the lung: a new method combining morphometry with mathematical modeling for airway classification Anat Rec 2002 266 1 51 57 10.1002/ar.10032 11748571

[B35] West JB Regional differences in the lung Chest 1978 74 4 426 437 10.1378/chest.74.4.426 699656

[B36] Burden RL Faires JD Numerical Analysis. The Prindle, Weber & Schmidt Series in Mathematics 1993 Boston: PWS-Kent Pub. Co

[B37] Hill EP Power GG Longo LD Mathematical simulation of pulmonary O2 and CO2 exchange Am J Physiol 1973 224 4 904 917 4698808 10.1152/ajplegacy.1973.224.4.904

[B38] Graeser HJ Kim YG Crandall ED The effects of time-varying blood flow on diffusional resistance to oxygen transfer in the pulmonary capillaries Biophys J 1969 9 9 1100 1114 10.1016/S0006-3495(69)86438-6 5807219 PMC1367546

[B39] Lindstedt SL Schaeffer PJ Use of allometry in predicting anatomical and physiological parameters of mammals Lab Anim 2002 36 1 19 10.1258/0023677021911731 11833526

[B40] Mateika JH Duffin J Changes in ventilation at the start and end of moderate and heavy exercise of short and long duration Eur J Appl Physiol 1992 65 234 240 10.1007/BF00705087 1396652

[B41] Reynolds WJ Milhorn HT Jr Transient ventilatory response to hypoxia with and without controlled alveolar PCO2 J Appl Physiol 1973 35 2 187 196 4723026 10.1152/jappl.1973.35.2.187

[B42] Wagner PD Gale GE Moon RE Torre-Bueno JR Stolp BW BW Saltzman HA Pulmonary gas exchange in humans exercising at sea level and simulated altitude J Appl Physiol 1986 61 1 260 270 3090012 10.1152/jappl.1986.61.1.260

[B43] Crocker GH Effects of Hypoxia, Hyperoxia, Hypercapnia and Elevated Carboxyhemoglobin Concentration on VO2max and Exercise Capacity in Goats. PhD Thesis 2010 University of California, Davis

[B44] Julien M Flick MR Hoeffel JM Murray JF Accurate reference measurement for postmortem lung water J Appl Physiol 1984 56 1 248 253 6693329 10.1152/jappl.1984.56.1.248

[B45] Huchon GJ Lipavsky A Hoeffel JM Murray JF Rebreathing lung tissue volume of sheep with normal and edematous lungs J Appl Physiol 1986 61 3 1132 1138 3759753 10.1152/jappl.1986.61.3.1132

[B46] Mundie TG Dodd KT Lagutchik MS Morris JR Martin D Effects of blast exposure on exercise performance in sheep J Trauma 2000 48 6 1115 1121 10.1097/00005373-200006000-00019 10866260

[B47] Gisolf J Wilders R Immink RV van Lieshout JJ Karemaker JM Tidal volume, cardiac output and functional residual capacity determine end-tidal CO2 transient during standing up in humans J Physiol 2004 554 Pt 2 579 590 14608002 10.1113/jphysiol.2003.056895 PMC1664761

[B48] MacFadden LN Chan PC Ho KH-H Stuhmiller JH A model for predicting primary blast lung injury J Trauma Acute Care Surg 2012 73 5 1121 1129 10.1097/TA.0b013e31825c1536 22914084

